# Capacity of the medullary cavity of tibia and femur for intra-bone marrow transplantation in mice

**DOI:** 10.1371/journal.pone.0224576

**Published:** 2019-11-07

**Authors:** Dieter Fink, Ulrike Pfeiffenberger, Tina Bernthaler, Sophie Schober, Kerstin E. Thonhauser, Thomas Rülicke

**Affiliations:** 1 Institute of Laboratory Animal Science, Department of Biomedical Sciences, University of Veterinary Medicine Vienna, Vienna, Austria; 2 Ludwig Boltzmann Institute for Hematology and Oncology, Medical University of Vienna, Austria; Emory University, UNITED STATES

## Abstract

Intra-bone marrow transplantation (IBMT) has been adapted for mouse models to improve the seeding efficiency of transplanted hematopoietic stem and progenitor cells. Commonly used injection volumes for IBMT into the tibia differ between 10 and 40 μL even though considerable amounts of injected cells leak into the blood circulation immediately after injection. Injection of 3 μL trypan blue into the tibia of dead BALB/c mice showed staining in large vessels of hind limbs, even without supporting circulation. We therefore tested the effective capacity of the medullary cavity of dissected tibiae and femora of different mouse strains by bioluminescence imaging after injection of luciferase expressing cells. Cell leakage was already observed at 3 μL of injection volume and the measured emission rate increased significantly when 5 and 10 μL of volume with the same cell concentration were injected. Surprisingly, increasing injection volumes containing constant cell amounts resulted in comparable emission rates, suggesting a similar amount of leaked and absorbed cells independent of the injection volume. However, the absorption of a specific amount of injected cells could not be confirmed, as the ratio of leaked to absorbed cells was similar between IBMT that were performed with a constant injection volume containing either low or high cell amounts. In summary, for optimal cell transplantation via IBMT in mice we suggest to inject a high concentrated cell suspension with a maximum injection volume of 3 μL.

## Introduction

Xenotransplantation assay in mice is an important approach to investigate biology of the human hematopoietic stem and progenitor cells (HS/PCs). Currently, leukemic stem cells (LSCs) including their repopulation and differentiation capacity after transplantation into mouse models are of particular interest in the investigation of different forms of myeloid and lymphatic leukemia [1].

Donor cell transplantation in mice is mostly performed by intravenous (i.v.) injection into the lateral tail vein and injected cells must pass through the lung before they enter the systemic circulation. The great majority of transplanted cells are retained in the lung and other organs like the liver, kidneys and spleen [2–4]. This stem cell trafficking vastly reduces the number of cells entering the bone marrow (BM) and might trigger a qualitative selection of donor cell subpopulations.

IBMT in mice has been developed to improve the seeding of injected cells in their preferred engraftment site. The procedure of orthotopic transplantation is a challenging procedure and it is associated with severe impairments for the recipient animals [5]. However, the BM cavity provides the specialized hematopoietic microenvironment for stem and progenitor cells and the reported seeding efficiency after IBMT is significantly higher compared to i.v. injections [3;4;6–9]. Furthermore, new classes of human HS/PCs could be identified by IBMT which cannot home into the BM niche from circulation [10].

Intraosseously infused donor cells were frequently detected in the marrow of other non-injected long bones, indicating a migration via blood circulation of transplanted cells from the injected site [7]. The fact that the vast majority of cells injected via the intraosseous route enter the systemic circulation implies that commonly used injection volumes may be excessive and unnecessary. The most frequently used injection volume for tibia is 10 μL, [7;10], however, volumes of 25 μL, 30 μL and even 40 μL have also been reported [6;8;9;11].

**Fig 1:**
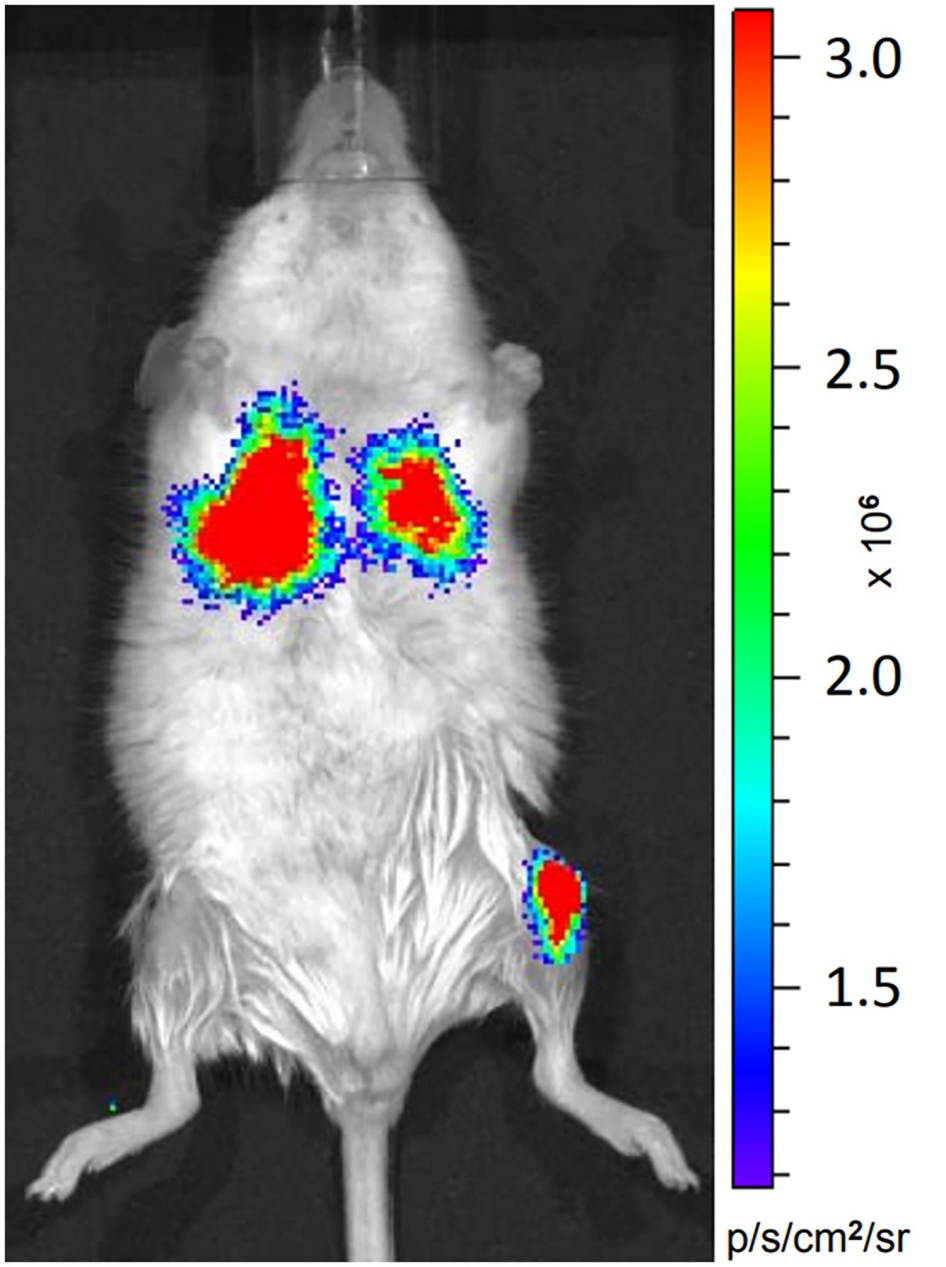
*In vivo* imaging of an adult BALB/c mouse. A cell suspension of 10 μL containing 200’000 luciferase expressing MDA-MB-231-luc were injected into the left tibia followed by an i.p. injection of 200 μL D-luciferine solution. The mouse was anaestetized by Isoflurane (2% in O_2_) and bioluminescence signals are detected in the area of the left tibia (injection site) and both lobes of the lung. Settings for *in vivo* imaging (Xenogen IVIS 50, PerkinElmer): medium binning, FOV7, exposure time 1 minute, bioluminescence was measured as normalized total flux (photons/s/cm2/sr) 12 min after IBMT.

**Fig 2:**
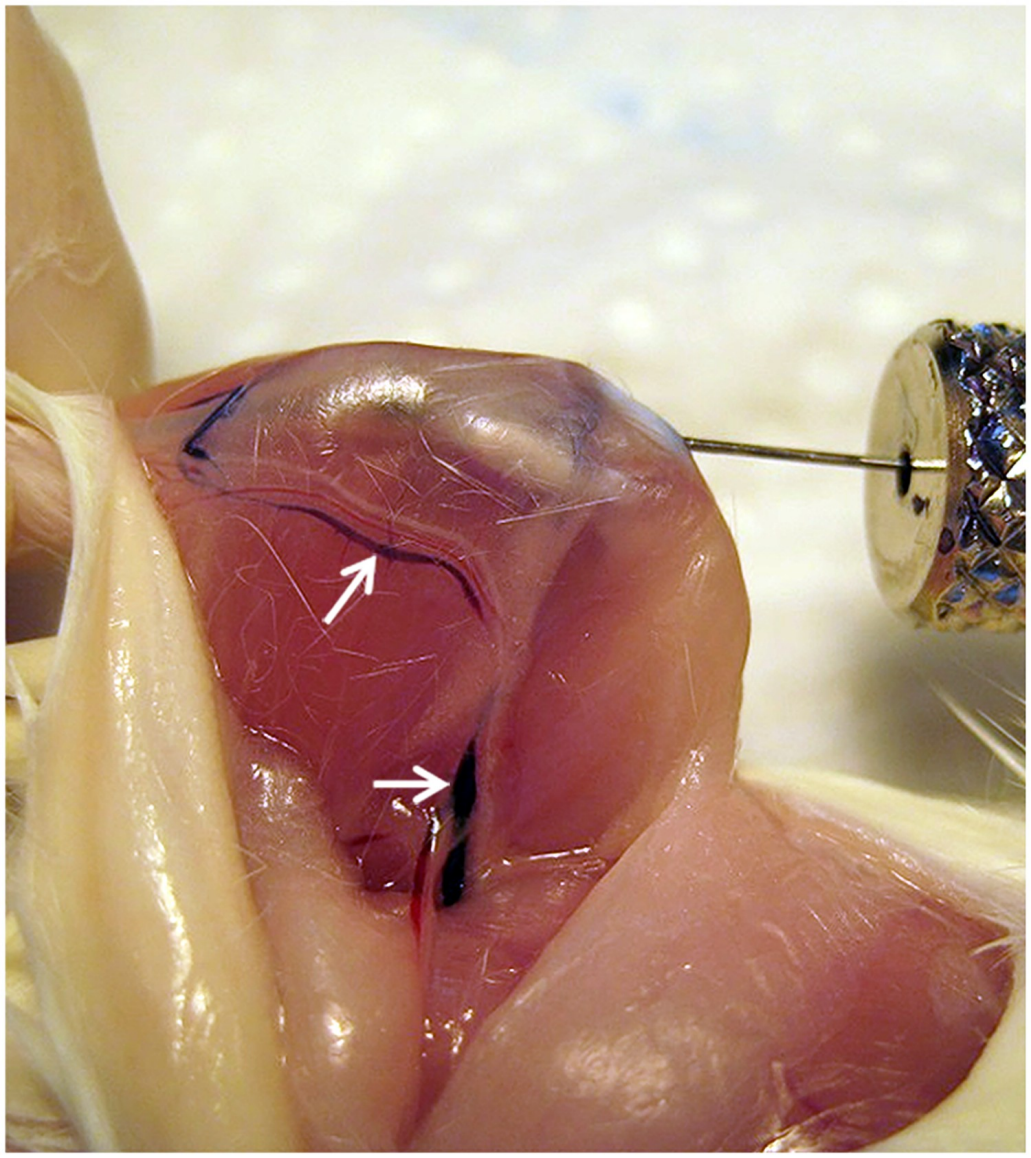
*In situ* IBMT of 3 μL trypan blue into the tibia of a dead BALB/c mouse. The skin of the right hind limb was carefully removed without harming anatomical structures. 3 μL trypan blue were injected into the tibia according to the standard procedure [5]. Staining becomes imediately visible in the medial v. saphena medialis and the v. femoralis (arrows).

In a previous study using IBMT in mice we decided to reduce the injection volume to 5 μL [5]. In preparation for the experiment we investigated the deposition of intraosseously injected cells and tested the most commonly used volume of 10 μL with luciferase expressing cells. Bioluminescence *in vivo* imaging revealed that most injected cells were deposited in the lung of the mouse immediately after injection (Fig 1). Additionally *in situ* test injections using trypan blue were performed into the tibia of freshly sacrificed mice to exclude any influence of the circulation. IBMT of 3 μL showed a clear injection volume transgression by staining in large vessels (*v*. *femoralis* and *v*. *saphena*) of skinned hind limbs (Fig 2). In order to save scarcely available patient donor cells, we here intended to define the capacity of the medullary cavity of long bones and the appropriate volume of cell suspensions for IBMT in adult mice of different mouse strains. Although the tibia is the preferred bone for IBMT, we also included the femur as a potential alternative in this investigation.

## Materials and methods

The study was approved by the institutional ethics committee of the University of Veterinary Medicine Vienna and an experimental license was granted under BMWF-68.205/0183-II/3b/2011. Adult mice of different strains in specific pathogen free (SPF) quality were housed in IVCs in a rodent facility (photoperiod 12L/12D, temperature 22.0°C ± 2.0°C). Food and water were available *ad libitum* and the mice were fed with a commercial regular mouse diet (ssniff Spezialitäten GmbH, Germany). Three mice were maintained per cage (Type IIL, Tecniplast, Italy) lined with bedding material (Lignocel^®^, heat treated, Rettenmaier KG, Austria) and enriched with nesting material (Pur-Zellin 4x5 cm; Paul Hartmann GmbH, Austria).

For the dissection of bones, animals were sacrificed by CO_2_ inhalation and cervical dislocation. The prepared tibiae and femora were pooled for sex and the two bones of the same type per animal were distributed to different treatment groups. Bones were stored in PBS (#806552, Sigma-Aldrich) on ice up to 2 hours until IBMT.

In order to quantify the amount of cells leaking from the bone during IBMT we utilized luciferase expressing MDA-MB-231-luc tumor cells and bioluminescence imaging. The cells were obtained from ATCC (U.K.), cultured at 37°C and 5% CO_2_ in DMEM media with high glucose and pyruvat (#D6546, Sigma-Aldrich), supplemented with 10% FCS (#10270106, Gibco) and 2 mM Glutamin (#G8541, Sigma-Aldrich). For harvesting, the cells were rinsed twice with PBS, trypsinized (0.05% Trypsin-EDTA, #25300054, Gibco), re-suspended in culture media, centrifuged at 1000 rpm, re-suspended in ice-cold PBS at 60 or 40 million per mL (cell stock solution), aliquoted in 2 ml reaction tubes à 10 or 20 μL depending on the injection volume and kept on ice until injection.

For a first assessment of the capacity of the medullary cavity we used adult BALB/cAnNCrl mice (3 females and 3 males, ages 3–4 months, body mass 25–30 g) for preparation of long bones, i.e. 12 tibiae and 12 femora. We tested three different injection volumes (3, 5, or 10 μL) of the prepared cell suspension using a 10 μL Hamilton syringe (700 series, needle size 30G). Each volume group consists of 4 femora or 4 tibiae, two bones from each sex. Each bone was injected once. The leaked cells were collected individually for each bone in a 24-well plate containing 200 μL D-luciferin solution per well (500 μg D-luciferin per mL in PBS, #122799, Perkin Elmer).

**Fig 3:**
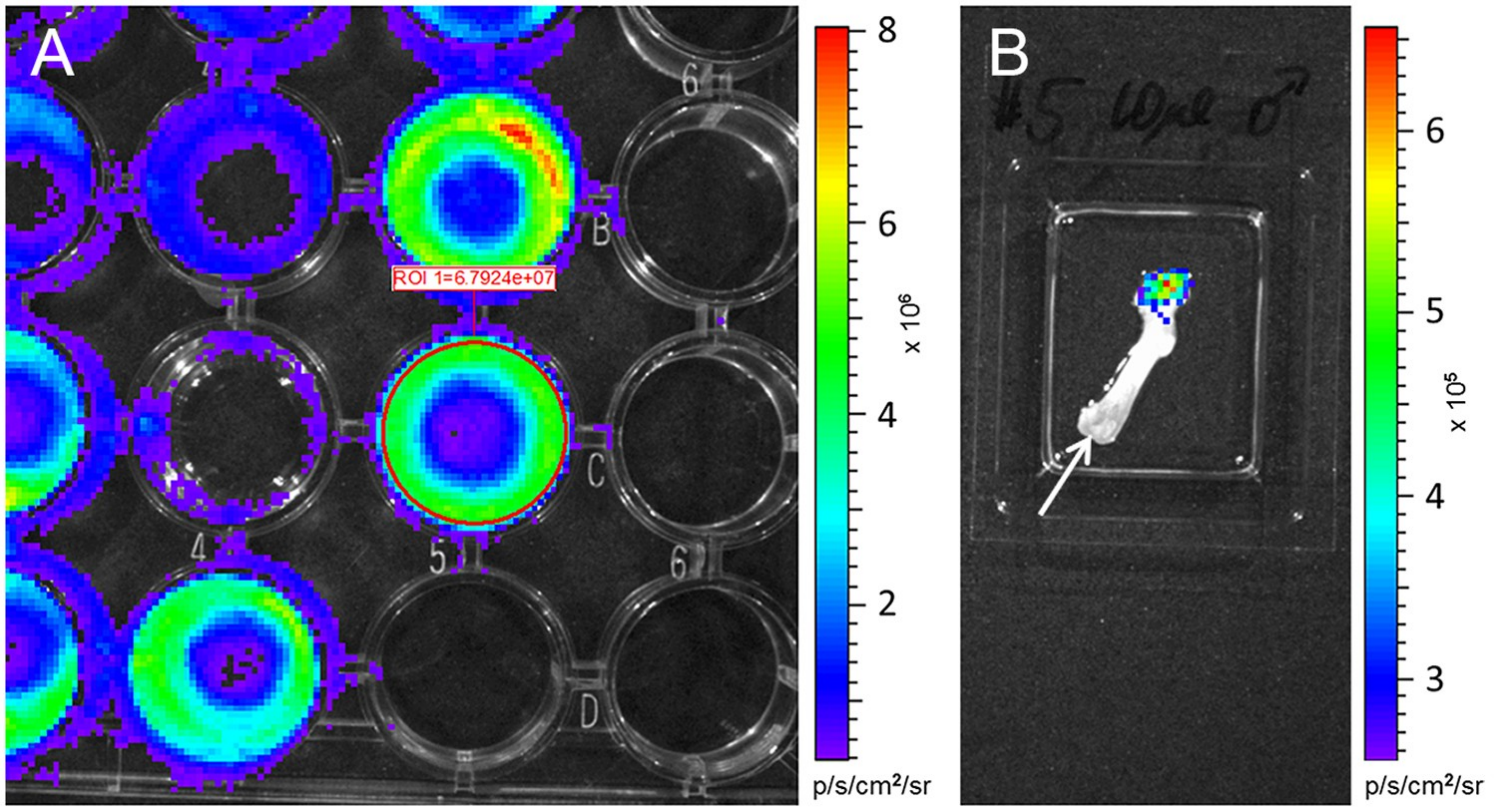
Measurement of bioluminescence signals of MDA-MB-231-luc cells leaking during IBMT from dissected long bones. (A) Emission of cells collected in a 24-well containing 200 μL D-luciferin solution after intraosseous injection of 10 μl cell suspension (~ 600’000 cells) into a femur. A ROI (region of interest) was placed over the surface area of the desired well and bioluminescence was measured as normalized total flux (photons/s/cm2/sr). (B) Control measurement for an injected femur in a separate well with 200 μL D-luciferin solution. Emission of remaining cells after rinsing the injected bone is visible oposite to the drilling tunnel. Arrow points to the injection site.

The *ex situ* IBMT was conducted according to our refined *in vivo* IBMT for tibia and femur [5]. Briefly, a tunnel was drilled through the joint surface of the knee using a needle (Braun^®^, 30Gx12 mm). After gently removing the drilling needle the injection needle was carefully inserted into the opening of the BM cavity and the defined volume of cell suspension was slowly injected into the medullary canal. Aspiration was avoided to minimize destruction of the local vasculature. Cells leaking during injection from the BM cavity via trans-cortical channels were collected in a well containing the D-luciferin solution. After injection and before the needle was slowly withdrawn, the part of the bone opposite to the drilling tunnel was rinsed to collect all cells from the outside surface of the bone in the D-luciferin solution. For control, the bone was then placed in a separate well containing D-luciferin solution. Two minutes after IBMT, light emission was measured for leaked cells of each injected bone by using an *in vivo* imager (Xenogen IVIS 50, Perkin Elmer) with the following settings: binning medium, FOV7, exposure time 10 s (Fig 3A). Afterwards the rinsed bone was measured for emission to confirm the almost complete collection of leaked cells (Fig 3B).

In order to evaluate the transferability of results obtained with BALB/c to other mouse strains, we tested tibiae and femora of males from NSG (NOD.Cg *Prkdc*^*scid*^ *Il2rg*^*tm1Wjl*^ /SZJ) mutants (4 males, age 3 months, body mass 31.0 g—32.0 g) and RjOrl:SWISS outbred mice (4 males, age 4 months, body mass 34.5 g—42.5 g), respectively. Mice of both strains are bred in our own animal facility. The preparation of MDA-MB-231-luc tumor cells and bioluminescence imaging was the same as applied for the previous experiment with the exception of a reduced cell concentration of 40 million per mL for the cell stock solution and the use of 500 μL D-luciferin solution per well in a 4-well plate (#176740, Thermo Fisher Scientific Thermo Fisher Scientific) for measuring the light emission of leaked cells. Additionally, bioluminescence imaging was performed on an IVIS Lumina S5 (PerkinElmer) with the following settings: binning 16, FOV10, exposure time 10 s. Groups of 4 tibiae and 4 femora per strain were injected either with 3 or 5 μL cell suspensions. We did not apply the 10 μL volume since the first experiment revealed this volume to be excessive and unnecessary.

To investigate the impact of cell concentrations on cell leakage after IBMT we injected (i) different volumes of cell suspensions with constant cell amounts and (ii) a constant injection volume with different cell amounts. For (i) 3, 5, and 10 μL injection volumes containing the same amount of cells (120’000) were prepared. The cell stock solution was diluted to 24 and 12 million cells per mL, aliquoted, and kept on ice until injection. For each volume group we used four tibiae or four femora isolated from six male NSG mice (ages 4–5 months, body mass 31.1–36.2 g).

For (ii) constant 3 μL injection volumes with different cell concentrations were prepared. The 40 million cells per mL were diluted to (rounded off) 20, 6.67, and 1.67 million cells per mL, which means that 120’000, 60’000, 20’000, and 5’000 cells were injected, respectively. For each volume group we used three tibiae or three femora isolated from six NSG males (age 2.5 months, body weight 26.4 g– 32.1 g).

To compare cell leakage and light emission in the different experimental groups, we applied nonparametric tests using IBM SPSS Statistics 24.

## Results and discussion

The BM of mice is described as a complex tissue with a high density of endothelial cells and mesenchymal stromal cells [12]. To define the maximum capacity of the medullary cavity of mice as recipients for intraosseous cell transplantation we injected in a first attempt three different volumes of cell suspension with the same concentration of 60 million cells per mL into femora and tibiae of female and male BALB/c mice. Since no significant sex differences in emission rates were detected (Mann-Whitney U Test: U = 62, p = 0.833), the data of BALB/c females and males within the same volume group were pooled.

**Fig 4:**
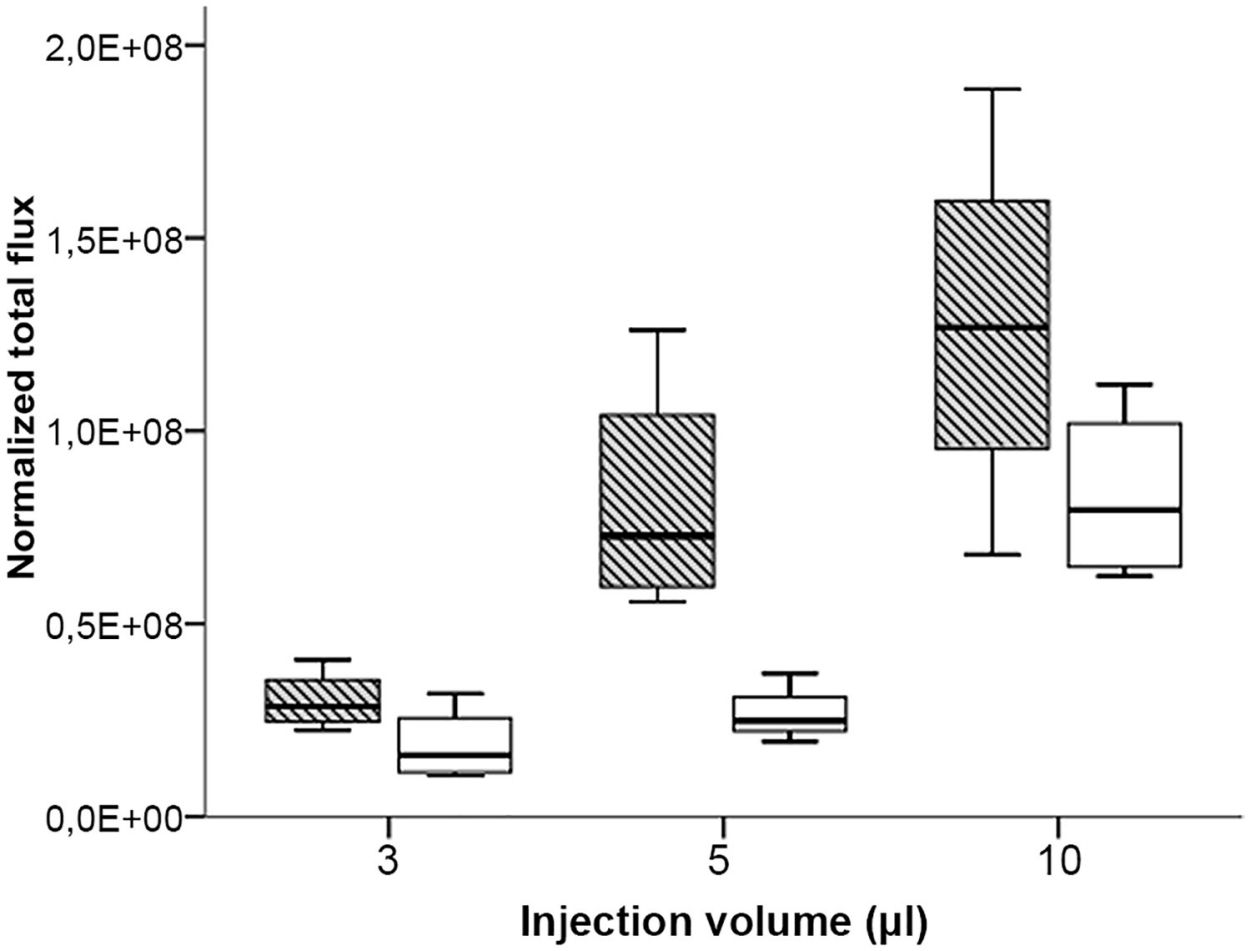
Quantitation of bioluminescence signals after injection of 3, 5 and 10 μL MDA-MB-231-Luc cell suspension (60 million per mL) into femur (striped box-plots) or tibia (open box-plots)of BALB/c mice. Results are depicted as boxplots, medians (lines in boxes), 25–75% iqr (boxes) and range from smallest to largest non-outliers. n = 4 per group (except for tibia 5 μl, n = 3). Bioluminescence was measured as normalized total flux (photons/s/cm2/sr).

Strong emission was already detected after the injection of 3 μL cell suspension (180’000 cells), indicating that even the smallest injection volume used in this experiment leads to a substantial release of injected cells from the medullary canal of both tested long bones. A restricted capacity of the BM cavity should result in an increasing amount of leaked cells after injection of larger injection volumes. Indeed, the emission values after injection of 5 and 10 μL suspension with the same cell concentration as used for the 3 μL volume increased significantly for tibia (Kruskal-Wallis Test: H = 7.85, p = 0.02) and femur (Kruskal-Wallis Test: H = 8.35, p = 0.015) (Fig 4).

**Fig 5:**
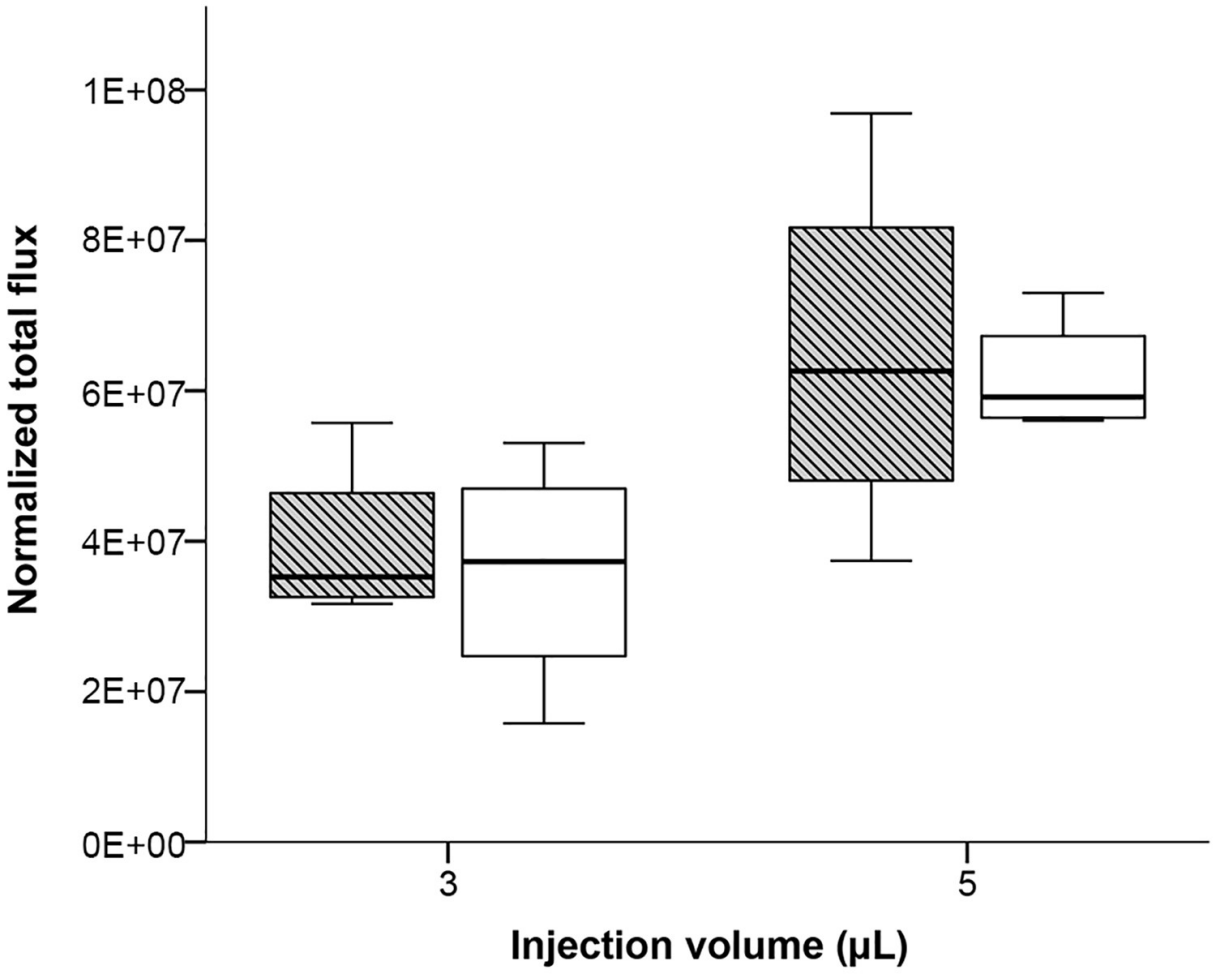
Quantitation of bioluminescence signals after injection of 3 and 5 μL of MDA-MB-231-Luc cell suspension (40 million per mL) into femur (striped box-plots) or tibia (open box-plots) of SWISS males. Results are depicted as boxplots, medians (lines in boxes), 25–75% iqr (boxes) and range from smallest to largest non-outliers. n = 4 per group. Bioluminescence was measured as normalized total flux (photons/s/cm2/sr).

Adult mice of the same age may have differently sized and shaped long bones depending on the strain. The growth curves of most important inbred strains like C57BL/6, C3H or DBA/2 provided by commercial breeders are very similar to BALB/c, suggesting comparable sizes of bones and marrow cavities. To demonstrate that the observed results from BALB/c mice are transferable to other mouse strains, we selected NSG and SWISS mice to assess the capacity of bone marrow cavities of medium- and large-sized mouse strains. Since the injection volume of 10 μL is definitely too high for IBMT in mice, only 3 μL and 5 μL suspension volumes were injected. The concentration of the cell suspension was slightly reduced to improve the injection performance. Cell leakage after injection of bones isolated from SWISS outbred mice significantly increased when the injection volume was raised from 3 μL to 5 μL in tibia (Mann-Whitney U Test: U = 16, p = 0.029). For femur a marginally non-significant trend was observed (Mann-Whitney U Test: U = 15, p = 0.057) (Fig 5). Although SWISS male mice used in this experiment have a much higher body weight than BALB/c (average body mass 38.6 g compared to 27.5 g) we observed substantial cell leakage even at the smallest volume of 3 μL.

**Fig 6:**
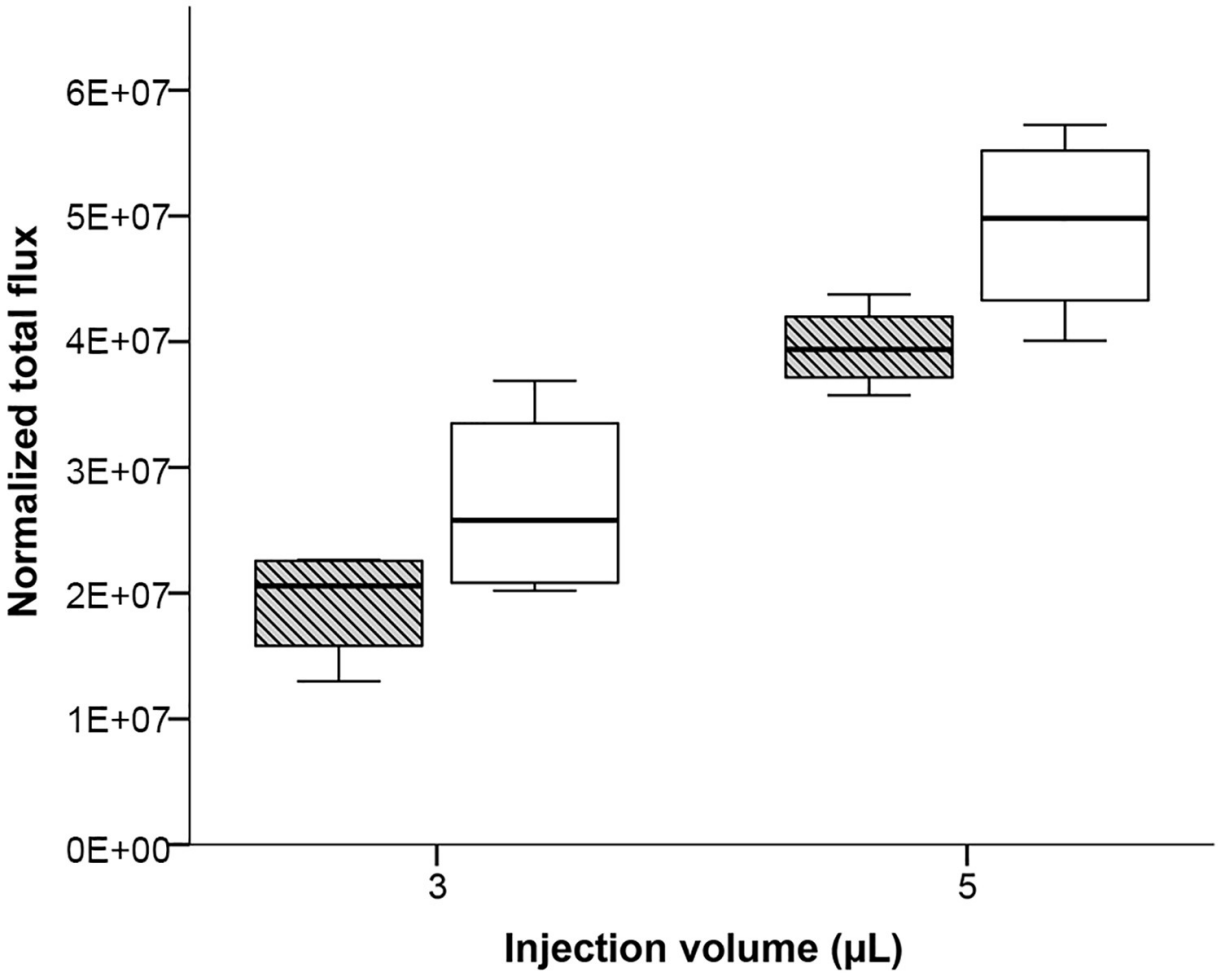
Quantitation of bioluminescence signals after injection of 3 and 5 μL of MDA-MB-231-Luc cell suspension (40 million per mL) into femur (striped box-plots) or tibia (open box-plots) of NSG males. Results are depicted as boxplots, medians (lines in boxes), 25–75% iqr (boxes) and range from smallest to largest non-outliers. n = 4 per group. Bioluminescence was measured as normalized total flux (photons/s/cm2/sr).

NSG and NSG related mouse strains are the most often used mutant models for xenotransplantation studies. The injection of 3 μL and 5 μL volume in both long bones of male NSG mice (average body mass 31.5 g) resulted similarly in a significantly increased emission rate for femur (Mann-Whitney U Test: U = 16, p = 0.029) and tibia (Mann-Whitney U Test: U = 16, p = 0.029) (Fig 6). Together, the results after IBMT of different injection volumes with the same cell concentration into femur and tibia of BALB/c, SWISS and NSG mice indicate that an injection volume of 3 μL exceeds the capacity of the bone marrow cavity measured by the emission of leaked luciferase expressing cells and that leakage increases with higher injection volumes.

Although marginally non-significant (Mann-Whitney U Test: U = 34, p = 0.051), the generally higher emission for the femur groups points to higher cell leakage from the femur in BALB/c compared to the tibia (Fig 4). In contrast, the results were reversed for NSG with a non-significant slightly higher emission for injected tibiae in this strain (Mann-Whitney U Test: U = 43, p = 0.279; Fig 6). In SWISS mice we observed no differences regarding the emission of leaked cells after IBMT between both tested long bones (Mann-Whitney U Test: U = 33, p = 0.916; Fig 5).

**Fig 7:**
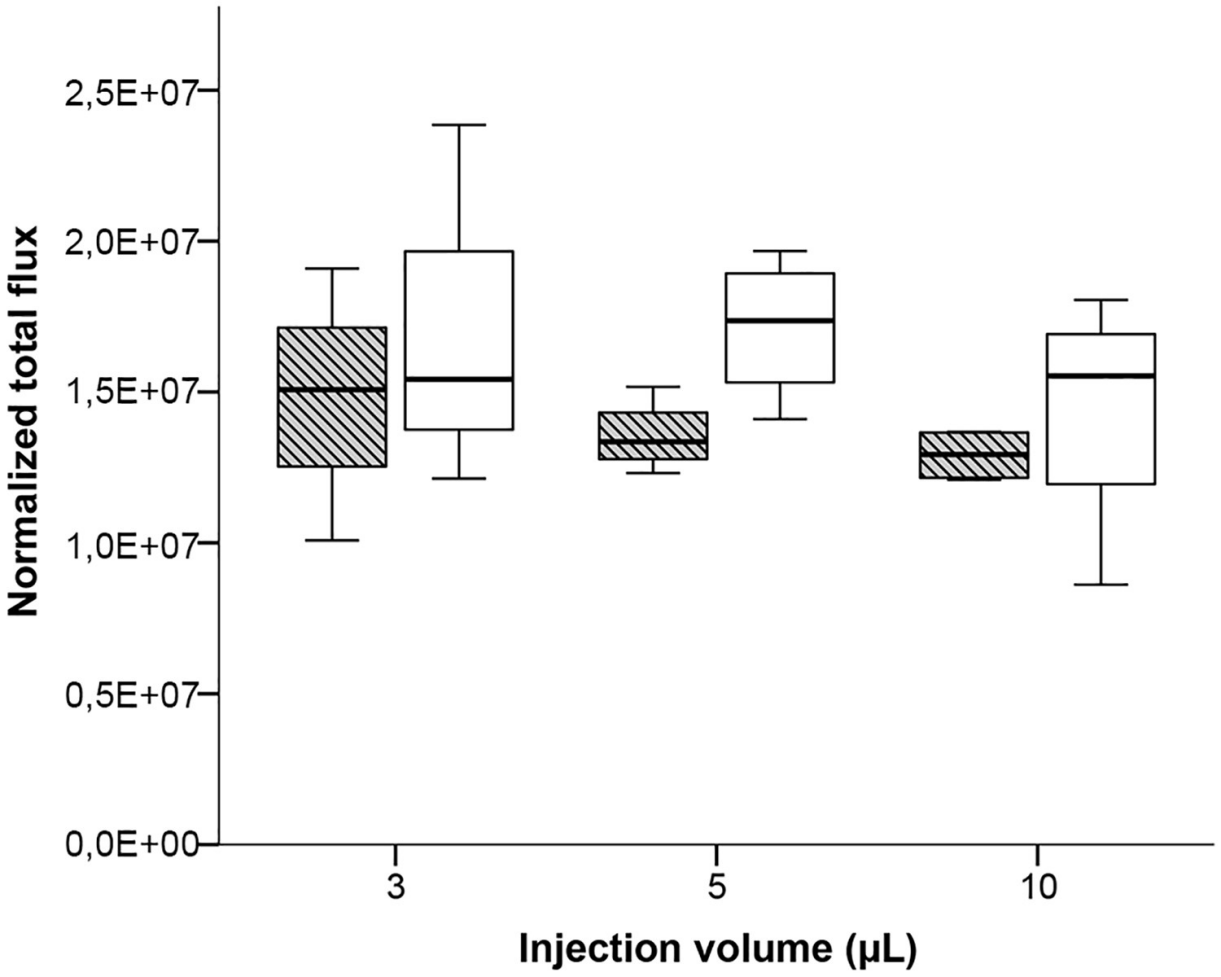
Quantitation of bioluminescence signals after injection of 3, 5 and 10 μL of MDA-MB-231-Luc cell suspension containing the same cell amount (120’000) into femur (striped box-plots) or tibia (open box-plots) of NSG males. Results are depicted as boxplots, medians (lines in boxes), 25–75% iqr (boxes) and range from smallest to largest non-outliers. n = 4 per group. Bioluminescence was measured as normalized total flux (photons/s/cm2/sr).

**Fig 8:**
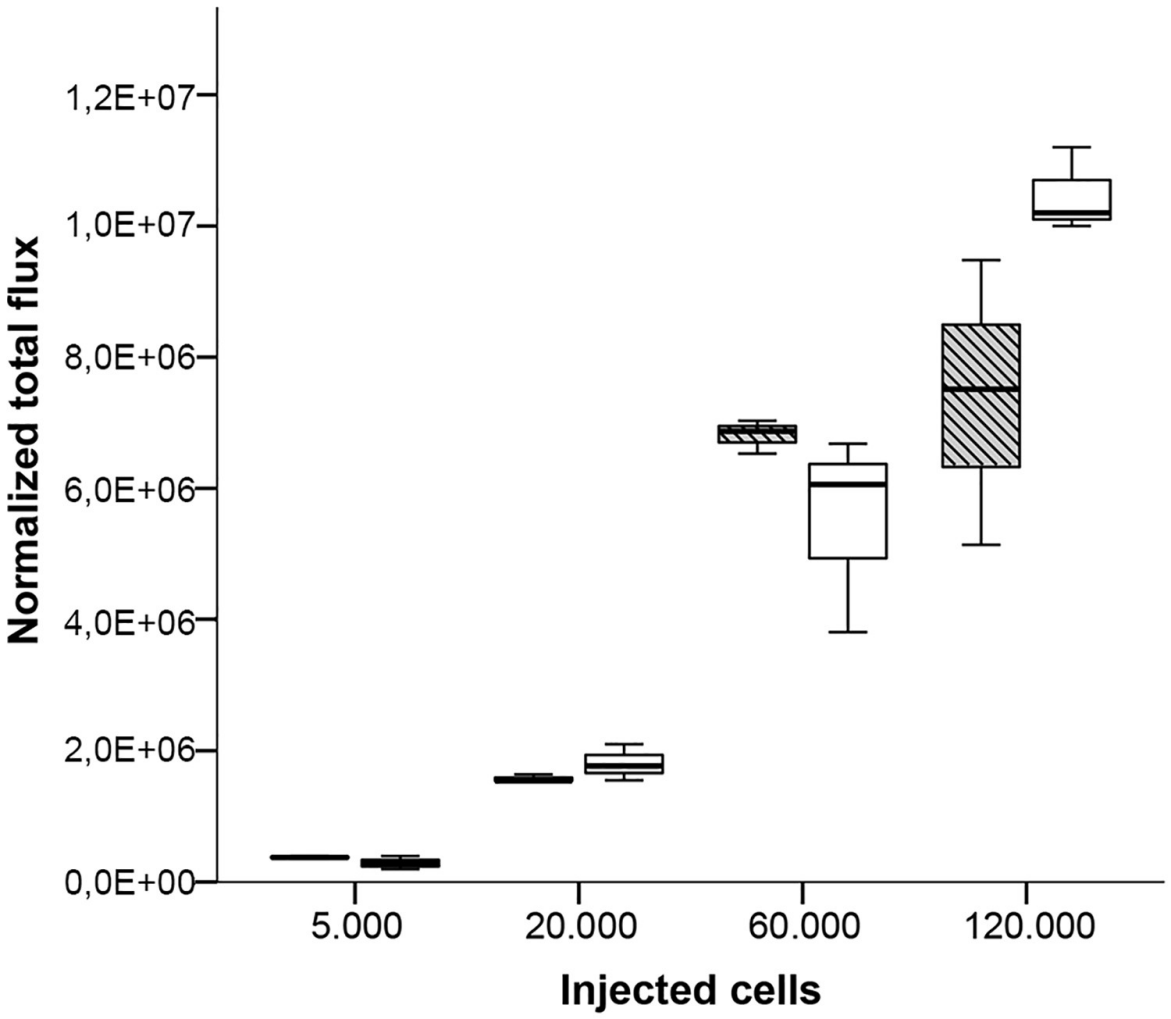
Quantitation of bioluminescence signals after injection of a constant volume of 3μL containing different cell amounts of MDA-MB-231-Luc cell suspension into femur (striped box-plots) or tibia (open box-plots) of NSG males. Results are depicted as boxplots, medians (lines in boxes), 25–75% iqr (boxes) and range from smallest to largest non-outliers. n = 3 per group. Bioluminescence was measured as normalized total flux (photons/s/cm2/sr).

The administration of increasing injection volumes containing a constant cell concentration per mL results in an increasing amount of transferred cells. Therefore, we aimed to test whether the total amount of injected cells or the injection volume has an impact on the absorption rate of injected cells in the BM. To test this, we first injected 3, 5 and 10 μL cell suspension containing the same cell amount of 120’000 cells into both long bones of male NSG mice (average body mass 33.1 g). The dilution of the stock solution resulted in decreasing cell concentrations per mL cell suspension from 3 μL to 10 μL injection volumes (40 million, 24 million and 12 million cells per mL). Assuming that the capacity of the medullary cavity is limited to a specific injection volume, the amount of leaked cells for the three injection volumes should nevertheless result in an increase of measured emission. Surprisingly, the emission rates of leaked cells were similar between the three injection volumes in both femur (Kruskal-Wallis Test: H = 1.515, p = 0.469), and tibia (Kruskal-Wallis Test: H = 1.192, p = 0.551) (Fig 7). A similar number of leaked cells, however, also suggest a similar number of cells absorbed in the BM independent of the injection volume. Assuming a restricted capacity of the BM cavity of less than 3 μL injection volume as shown for trypan blue injection (Fig 2), the question arises whether the BM can absorb a specific amount of injected cells. To investigate this possibility we injected four different amounts of cells (5’000, 20’000, 60’000 and 120’000) suspended in the same injection volume of 3 μL in male NSG mice (average body mass 29.6 g). To make a rough estimate about the leaked cells, the same cell suspensions were directly injected into the well with 500 μL D-luciferin solution and the emission rate was quantified. Interestingly, emission was measured even after injection of only 5’000 cells suspended in 3 μL, revealing cell leakage at the lowest cell amount. The emission intensity increased with the injected cell amount (Fig 8). However, the relative difference in mean emission rates between the leaked cells after injection and the corresponding cell suspension without injection was comparable between the lowest and the highest cell amount (Table 1). These results suggest that it is the injection volume and not the cell amount that mainly determines cell leakage after an IBMT procedure.

**Table 1:** Overview of the mean emission rates (normalized total flux) between leaked cells after 3 μL IBMT in male NSG mice and the corresponding cell suspension without IBMT. n = 3 per bone and injected cell amount.

bone	# injected cells	mean flux of corresponding cells	mean flux after IBMT injection	absolute difference in mean flux	relative difference in mean flux in %
femur	120.000	1.42E+07	7.38E+06	6.82E+06	48.04
tibia	120.000	1.42E+07	1.05E+07	3.73E+06	26.27
femur	60.000	8.52E+06	6.81E+06	1.71E+06	20.07
tibia	60.000	8.52E+06	5.52E+06	3.00E+06	35.22
femur	20.000	2.04E+06	1.57E+06	4.66E+05	22.85
tibia	20.000	2.04E+06	1.81E+06	2.34E+05	11.46
femur	5.000	5.22E+05	3.76E+05	1.46E+05	28.00
tibia	5.000	5.22E+05	2.89E+05	2.33E+05	44.66

For IBMT the mice are usually exposed to a unique sublethal dose of total body irradiation about 24 hours prior to injection. The treatment will not only damage hematopoietic stem cells and downstream progenitors but also the BM stroma. Although the impact of irradiation on the capacity of the medullary cavity was not investigated here we do not expect relevant differences between isolated bones used in our study or bones from irradiated mice at the time point of transplantation. It has been shown that significant reductions in the absolute number of BM stroma cells will only be detectable weeks after the irradiation exposure of mice [13].

Recently, a dense network of trans-cortical vessels (TCVs) in the tibia of mice has been described as largely responsible for the extensive blood flow into and out of long bones. Venous TCVs are suggested to facilitate the egress of bone marrow cells and could also provide the network of routes for transplanted cells to leave the bone marrow cavity [14]. Whether the observations of our study are generally applicable to other cell types, especially to stem cells, as well as to IBMT *in vivo* condition, have to be further investigated.

Compared to i.v. injection, IBMT provides superior seeding efficiency of HS/PC/LSCs [3;4;6–9]. However, IBMT is also associated with increased distress and impact on the wellbeing of the treated animals, especially when the more stressful tibia injection is applied [5]. To improve the harm-benefit ratio of the IBMT technique and to optimize the potential to save limited patient donor cells, a lowered injection volume should be fully utilized. A high cell concentration in the injection volume will support a high cell amount resorbed in the BM cavity. However, the final concentration of the cell suspension is restricted to ensure an injectable consistency for a 30G needle that should be used to refine the procedure. Taking the limited capacity of the bone marrow cavity and the technically challenging procedure of IBMT into account we recommend a maximum of 3 μL cell suspension as injection volume for IBMT into the tibia and femur of adult laboratory mice.
